# Interferometric Near-field Fano Spectroscopy of Single Halide Perovskite Nanoparticles

**DOI:** 10.1021/acs.nanolett.4c04491

**Published:** 2024-11-28

**Authors:** Jinxin Zhan, Tom Jehle, Sven Stephan, Ekaterina Tiguntseva, Sam S. Nochowitz, Petra Groß, Juanmei Duan, Sergey Makarov, Christoph Lienau

**Affiliations:** † Institut für Physik, Carl von Ossietzky Universität, 26129 Oldenburg, Germany; ‡ Department of Nanophotonics and Metamaterials, ITMO University, St. Petersburg 197101, Russia

**Keywords:** Fano interference, MAPbI_3_ nanoparticle, near-field scattering-type spectroscopy, time-domain near-field spectroscopy

## Abstract

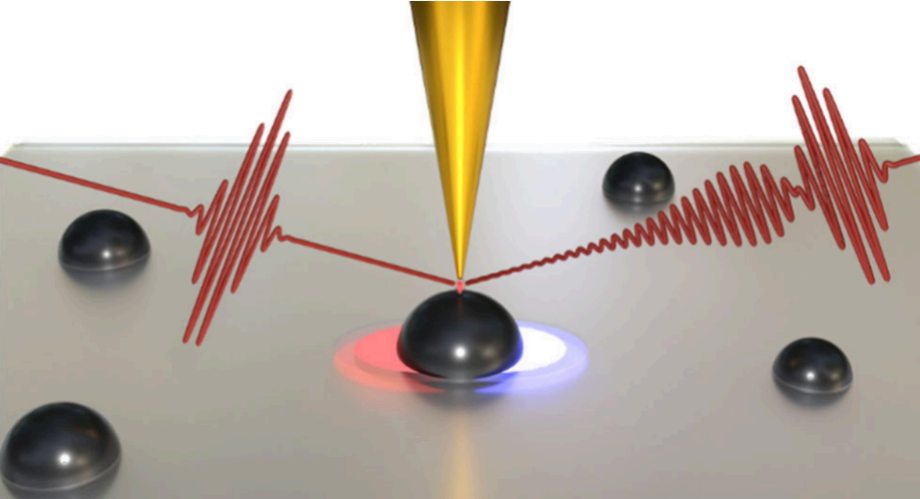

Semiconducting halide perovskite nanoparticles support Mie-type resonances that confine light on the nanoscale in localized modes with well-defined spatial field profiles yet unknown near-field dynamics. We introduce an interferometric scattering-type near-field microscopy technique to probe the local electric field dynamics at the surface of a single MAPbI_3_ nanoparticle. The amplitude and phase of the coherent light scattering from such modes are probed in a broad spectral range and with high spatial resolution. In the spectral domain, we uncover a Fano resonance with a 2π phase jump. In the near-field dynamics, this Fano resonance gives rise to a destructive interference dip after a few femtoseconds. Mie theory suggests that the interference between electric quadrupole and magnetic dipole modes of the particle, with spectra affected by resonant interband absorption of MAPbI_3_, lies at the origin of this effect. Our results open up a new approach for probing local near-field dynamics of single nanoparticles.

Metallic, semiconducting and dielectric nanoparticles form outstanding tools for localizing light on the nanoscale. Their optical shape resonances confine light in certain localized modes and spectral regions. The resonances are largely tunable by varying the size, shape or composition of the particle. Such particles find broad applications in nanosensing, nonlinear optical switching, photocatalysis, and nanophotonics.^1^ Dielectric nanoparticles with high refractive index (*n* > 2) recently attracted much attention since they provide the possibility to control light on a subwavelength scale with low losses and high-damage thresholds as compared with plasmonic particles.^2^ While their lowest order dipolar modes are spectrally rather broad, higher order electric and magnetic modes offer interesting opportunities for confining light in spectrally sharp shape resonances. In such nanostructures, the interplay between different magnetic and electric resonances is crucial to efficiently control the light matter interaction in single nanoparticles^3^ or metasurfaces.^4,5^

Halide perovskites of the MAPbX_3_ family, where MA denotes methylammonium (CH_3_NH_3_) and X stands for I, Br, or Cl, are promising candidates for optoelectronic device applications.^6^ MAPbX_3_ is a semiconductor with a chemically tunable bandgap, supporting excitonic states at room temperature, which allows for tuning the light emission from MAPbX_3_ nanoparticles over the entire visible spectrum (400–800 nm).^7^ Halide perovskites nanostructures with different shapes, e.g. nanowires,^8^ microplates,^9^ microspheres,^10^ and nanocubes^11^ can be fabricated by simple chemical methods, providing excellent accessibility of electric- and magnetic-type resonances including higher order resonances.

The optical spectra of MAPbX_3_ nanoparticles show two distinct types of shape resonances. Below the MAPbX_3_ bandgap, far field light couples to the regular Mie resonances of a high-refractive index dielectric nanoresonator. A destructive interference in backward scattering direction between different of these Mie modes gives rise to distinct Kerker resonances.^12−16^ Around the bandgap, the optical excitations of the semiconductor material, excitons and electron–hole pairs, are efficiently coupled to these dielectric Mie resonances, making such nanoparticles interesting for ultrafast optical switching and nanoscale lasing. This coupling leads to distinct hybrid Fano resonances^17,18^ and results in rather complex single particle scattering spectra.^17^ These spectra display the dipolar and quadrupolar shape resonances of a purely dielectric nanoparticle^15,16^ but with a line shape that is significantly affected by the onset of interband absorption in the semiconducting medium. As such, one expects complex spatiotemporal near-field dynamics on few-femtosecond time scales which have not been studied so far.

Much progress has recently been made in imaging nano-optical fields using a variety of quasi-stationary all-optical and electron-based spectroscopy techniques,^19^ such as scanning near-field optical microscopy (SNOM),^20^ photon-induced force microscopy,^21^ cathodoluminescence,^22^ electron energy loss spectroscopy,^23^ photon-induced near-field electron microscopy (PINEM)^24−27^ or photoelectron emission microscopy.^28^ Despite this progress, techniques that resonantly probe the near-field dynamics of single nanoparticles in space and time are still in an early stage of their development, mainly because of the difficulty in isolating the weak near-field scattering from background contributions. In particular, the near-fields of perovskite nanoparticles and their dynamics have not yet been explored and information about Fano resonances has so far been obtained less directly from far-field experiments.^17^

Here, we describe and demonstrate an interferometric scattering-type near-field microscopy technique to probe the time dynamics of local optical fields around single MAPbI_3_ nanoparticles. A destructive interference that occurs a few femtoseconds after optical excitation is the signature of a Fano resonance with a characteristic 2π phase jump in the spectral domain. We show that the interference between electric quadrupole and magnetic dipole and modes of the particle, with spectral shape significantly affected by resonant interband absorption of MAPbI_3_, lie at the origin of this Fano resonance.

We investigate MAPbI_3_ perovskite nanoparticles with diameters in the range of a few hundred nanometers, fabricated by laser ablation of a perovskite thin film on a 125 μm thick cover glass slip (see Sections 1 and 2 of the Supporting Information).^7,17,29^ To probe the temporal dynamics of optical near-fields at the surface of single nanoparticles, we use a broadband near-field spectral interferometry setup depicted in Figure 1a. A few mW of linearly p-polarized light from a titanium:sapphire laser, delivering 6 fs pulses centered at 780 nm with a repetition rate of 80 MHz, are focused to a few-micron spot at the apex of a sharp scattering-type gold nanotaper using an all-reflective microscope objective. The taper is mounted in a tapping mode force microscope, modulating the tip–sample distance at frequency *f*. The light scattered from the tip is collected in back-reflection geometry and detected using an avalanche photodiode connected to a lock-in amplifier. This signal is then demodulated at different harmonics of the tip modulation frequency.^30^ Alternatively, the tip-scattered light (the signal field) is overlapped with a time-delayed replica of the incident laser (the reference field) on a broad-band beam splitter. The superposition of signal field ***E***_*S*_(ω) and linearly polarized reference field ***E***_*R*_(ω) is spectrally dispersed in a monochromator and recorded with a fast line camera, see Section 3 of the Supporting Information. The resulting spectral interferogram (SI)^31,32^ (Figure 1b) can be written approximately as
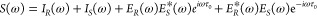
1where *I*_*R*_(ω) = |***E***_*R*_(ω)|^2^, *I*_*s*_(ω) = |***E***_*s*_(ω)|^2^, and *E*_*S*_(ω) = ***E***_*S*_(ω)·***n*** with ***n*** denoting the polarization direction of the reference. The time delay between signal and reference is τ_0_. Since the acquisition time of the camera is only 4.6 μs, the recorded SIs are unaffected by mechanical interferometer instabilities. Experimentally, we τ_0_ ≈ 1100 fs, giving a fringe spacing of 2 nm. At every position of the tip on the sample, we record a series of 60.000 SIs while periodically modulating the tip–sample distance. With a time interval of 4.6 μs this takes about 0.28 s at every pixel of a near-field image.

**Figure 1:**
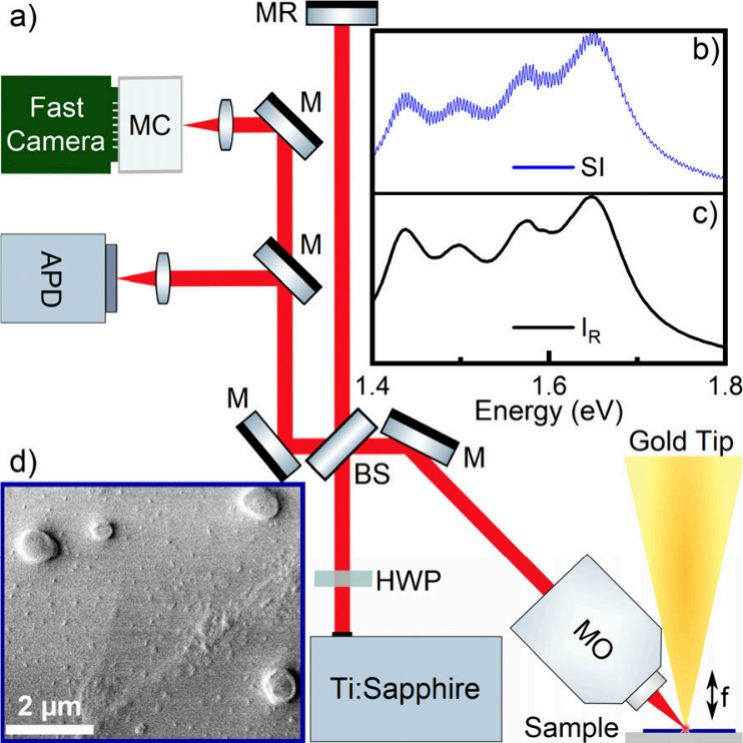
(a) Schematic setup of spectral interferometry scanning near-field spectroscopy (SI-SNOM). P-polarized Light from a 6 fs Ti:sapphire laser is focused onto the sample. Phase stable spectral interferograms between the light scattered from the sample and the laser light are recorded with a fast last line scan camera at a rate above the tip modulation frequency *f*. (b) Exemplary spectral interferogram. (c) Spectrum of the laser in the reference arm. (d) Scanning electron microscope image of single MAPbI_3_ particles fabricated by laser ablation.

Amplitude and spectral phase of the signal field can be obtained from a Fourier domain analysis of the SIs as described in Sections 4–6 of the Supporting Information. This provides the complex-valued spectral response function σ(ω) = *E*_*S*_(ω)/*E*_*R*_(ω) that connects the field that is scattered from the tip to the reference field. This response function still contains contributions from undesired background scattering from the tip shaft. We therefore extract this response function for each of the 60.000 SIs that are recorded at one tip position. Then, we perform a demodulation of these response functions at the n-th harmonic of the tip modulation frequency, in analogy to the lock-in analysis of the APD signals (eq (21) of the Supporting Information). Since the reference signal is much stronger than both the near-field and background scattering, these demodulated response functions of second and higher order are only sensitive to the near-field scattering, while background scattering is weak. Hence, these higher order demodulated spectra contain the complete information about the desired near-field response function σ_*NF*_(ω) that connects the reference field and the local field in the tip–sample junction. To optimize the signal-to-noise ratio, we identify the second-order demodulated response function with σ_*NF*_(ω) and do not consider the higher-order demodulate signals further. We now employ this new method to investigate near-field scattering from individual MAPbI_3_ particles.

A scanning electron microscope image of these particles is depicted in Figure 1d, showing irregularly shaped half-spherical particles with radii ranging from several tens to hundreds of nanometers. We start by mapping spatial near-field distributions of individual MAPbI_3_ particles using broadband optical excitation and spectrally integrated detection of the near-field using the demodulated APD signal. The topographic image of a typical area in the sample is shown in Figure 2a. We map several MAPbI_3_ particles with diameters ranging from 100 to 500 nm. The corresponding near-field image in Figure 2b shows the APD signal demodulated at the third harmonic of the tip modulation frequency. It is completely dominated by near-field scattering from the MAPbI_3_ particles while undesired background scattering is weak. For the smallest particles, the near-field images show a dipole-like field distribution at the particle surface, with the line connecting the two maxima oriented along the polarization direction of the incident laser. For the larger particles, we find, in addition to this dipolar pattern, a characteristic ring-like structure at the rim of the particle.

**Figure 2:**
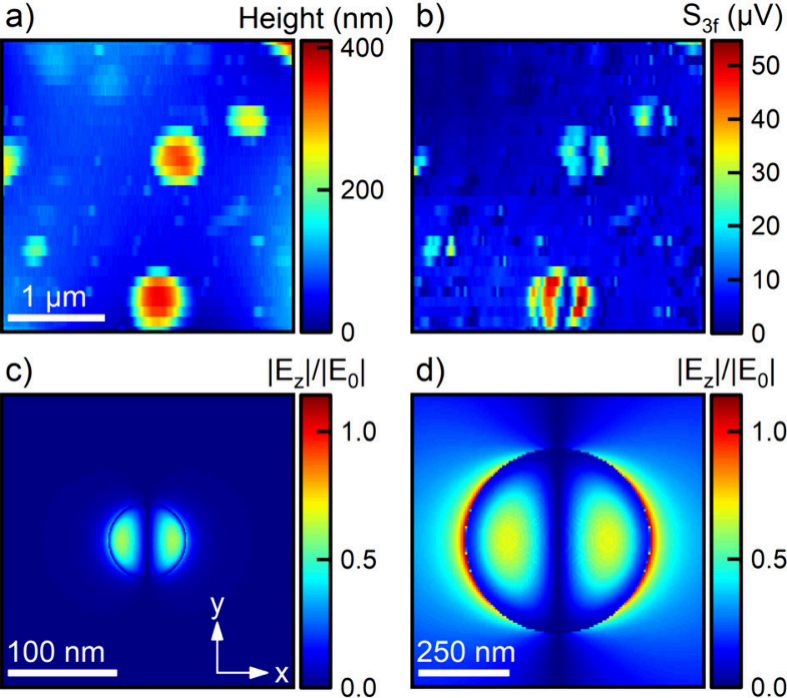
(a) Topography image of a 3 μm × 3 μm area of a sample with laser-ablated MAPbI_3_ nanoparticles on a glass coverslip. (b) Map of the optical near-field signal recorded for broadband, linearly p-polarized excitation of the sample with a 6 fs Ti:sapphire laser and detected using an avalanche photodiode (APD). The image shows the APD signal, demodulated at the third harmonic of the tip oscillation (*S*_3*f*_). For small particles, the images show a dipole-like field distribution, mapping primarily the z-component of the electric near-field on the nanoparticle surface. Larger particles show an additional ring-like structure near their rim. (c,d) Simulated map of the z-component of the local optical near-field pointing along the taper axis, summed over a spectral range from 650 to 950 nm, at the surface of MAPbI_3_ nanoparticles with radii of 50 nm (c) and 250 nm (d), respectively. The simulations reproduce the ring-like field at the rim of larger particles.

Finite difference time domain (FDTD) simulations of the near-field distributions for half-spherical MAPbI_3_ particles on a glass substrate confirm these observations if we assume (i) linearly x-polarized excitation at normal incidence and (ii) that the near-field images map the near-field component normal to the substrate surface, *E*_*NF,z*_, that points along the tip axis. Figure 2c,d shows the calculated field at a distance of 1 nm above the surface of the sample. For small particles with a radius of 50 nm (Figure 2c), the near-field image exhibits the same dipolar pattern, mainly confined to the inner rim of the particle, that is seen in experiment. For such small particles, the electric dipole (ED) and magnetic dipole (MD) modes can be excited in a broad spectral range.^17^ The near-field distributions of both modes are qualitatively similar. Both show dipolar pattern (Figure S5 of the Supporting Information) with the ED mode being confined to the inside of the sphere while the MD pattern is centered around the rim of the particle. Both dipole modes may contribute to the field distributions in Figure 2b, intuitively explaining the observed pattern for small particles. For larger particles, with diameters in the range of 150 nm or beyond, not only these dipole modes but also the electric and magnetic quadrupole modes, EQ and MQ, can be excited, resulting in more complicated field distributions. Since we are mapping the z-component of the optical near-field, the characteristic outer rim ring structure can be associated with the excitation of the EQ mode. For a spherical particle, a linearly polarized plane wave excites a quadrupole in the plane that is spanned by the wavevector and the polarization direction of the light. Light that is x-polarized thus excites a quadrupole in the *x*–*z*-plane, i.e, two dipoles pointing in opposite direction along x that are slightly displaced along z.^33^ When placed on top of this sphere, the tip mainly probes the field of the upper dipole, giving rise to the inner, dipole-like pattern in Figure 2d. Near the rim, the tip senses the field lines that are connecting the two stacked dipoles. This causes the characteristic field node that is seen in Figure 2b,d in between the two regions. This qualitative mode assignment forms a solid basis for analyzing spectrally resolved near-field maps presented in Figure 3.

**Figure 3:**
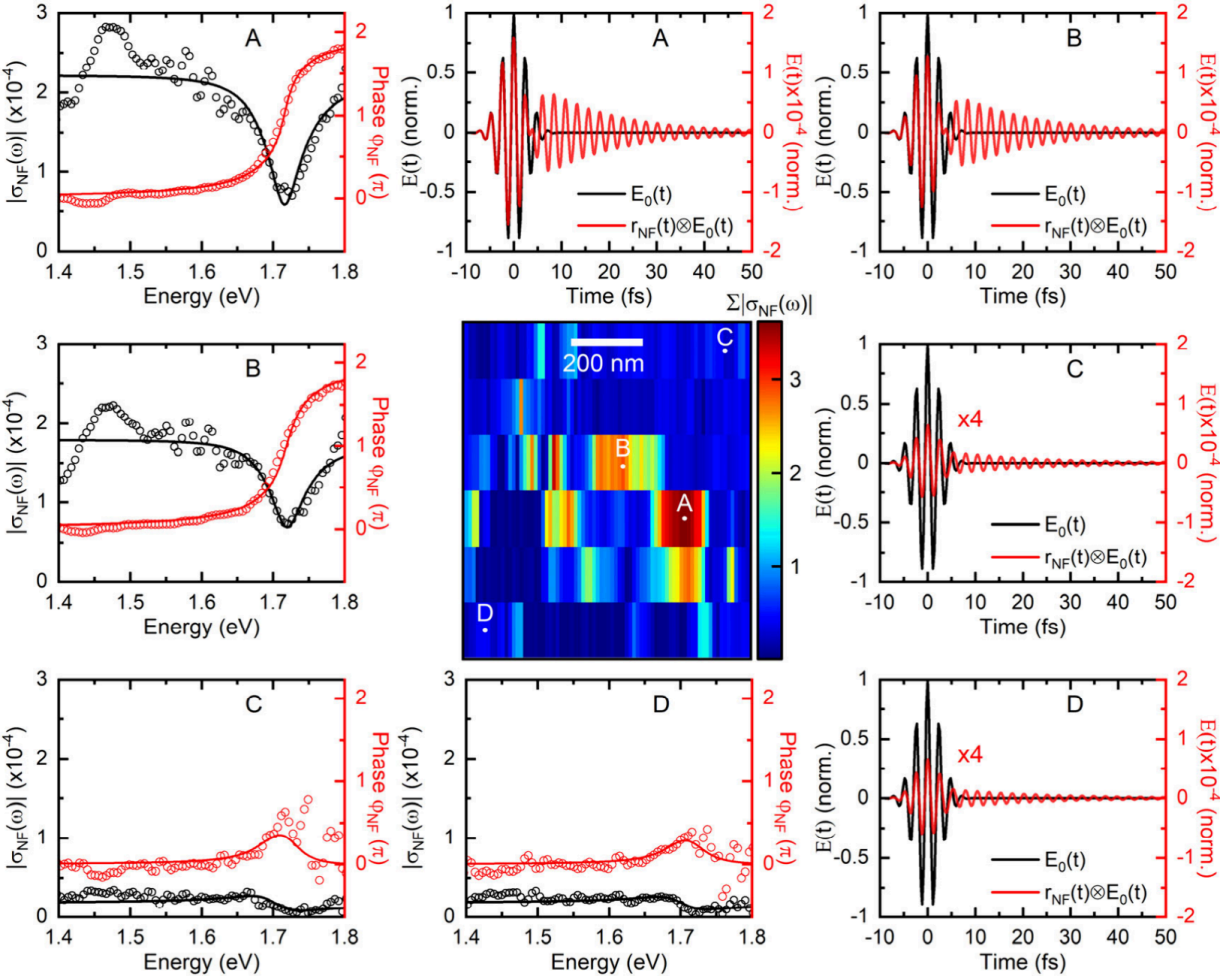
Near-field response functions σ_*NF*_(ω) and local near-field dynamics at the surface of a MAPbI_3_ nanoparticle reconstructed from spectral interferometry SNOM. The middle panel shows a spatial map of the spectrally integrated local near-field response functions recorded in the vicinity of a particle with a diameter of 500 nm. The left panels show the amplitude (black circles) and spectral phase (red circles) of σ_*NF*_(ω) at positions A-D at the sample surface. At A and B, a reduction in near-field scattering connected with a 2π phase jump is found around 1.72 eV. A Fano model is used to fit the results (solid curves). The right panels display the corresponding reconstructed near-field dynamics *E*_0_(*t*)⊗*r*_*NF*_(*t*) (red lines) at A-D, together with the bandwidth limited electric field profile of the excitation laser *E*_0_(*t*). The long-lived response at A and B reflects the excitation of the EQ mode while the characteristic dip around 3 fs arises from its Fano-type interference with the short-lived dipole modes. At C and D, the excitation of EQ is weak and the dynamics is dominated by the quasi-instantaneous scattering from the glass substrate.

To record amplitude and phase resolved local near-field spectra the tip is raster-scanned across an 800 nm by 900 nm area around a single MAPbI_3_ particle with a diameter of ∼500 nm. The step size is 6.6 nm in *x*-direction, along the polarization direction of the incident laser, and 180 nm in y. A map of the total, spectrally integrated field amplitude (center panel in Figure 3) shows again a pronounced dipole-shaped near-field enhancement at the surface of the particle. Evidently, the optical quality of the map is limited by the slow scan speed and coarse pixel size and the outer rim structure in Figure 2b cannot faithfully be resolved. At the positions of large field enhancement, e.g., positions A and B, the amplitude of the near-field response |σ_*NF*_(ω)| displays a pronounced dip at an energy of 1.72 eV. This dip is associated with an unusual phase jump of 2π, in stark contrast to the π phase jumps that are commonly seen in far field spectra. In addition, the spectra reveal a fainter second resonance around 1.45 eV. These two, comparatively narrow spectral resonances are readily assigned to the magnetic (1.45 eV) and electric (1.72 eV) quadrupole resonances of the particle. At positions C and D in Figure 3, the field enhancement is much reduced and we observe a slight dip in scattering amplitude and a small increase in phase at the EQ resonance. Since both amplitude and phase of σ_*NF*_(ω) are known in a rather broad spectral range, the local time-domain response at the tip position ***r***_*t*_ can directly be obtained by Fourier transformation *r*_*NF*_(*t*,***r***_*t*_) = *Re*(*F*(σ_*NF*_(ω,***r***_*t*_)). Experimentally, this response function is measured in a finite spectral range given by the bandwidth of the excitation laser, ranging, in our case, from 1.4 to 1.8 eV. Therefore, the reconstructed near-field dynamics *E*_*NF*_(*t,***r**_*t*_) = *E*_0_(*t*)⊗*r*_*NF*_(*t,***r**_*t*_) should be obtained by convoluting the response function with the bandwidth-limited time profile *E*_0_(*t*) = *Re*(*F*(|*E*_*R*_(ω)|) of the excitation laser. The reconstructed near-field dynamics *E*_*NF*_(*t,***r**_*t*_) at positions A-D are plotted in Figure 3 as red lines, together with *E*_0_(*t*) in black.

At positions C and D, the near-field dynamics mainly follow the field profile of the excitation laser since they are dominated by off-resonant light scattering from the substrate. Here, the local near-field is in phase with the driving field. This provides a reference for the zero-order phase of the optical near-field. A faint long-lived response, with an oscillation period of 2.4 fs (*ℏω*_*EQ*_*=*1.72 eV) and an exponential dephasing time of *T*2_*EQ*_ = 16 fs (*ℏγ* = *ℏ*/*T*2_*EQ*_= 42 meV) reflects the free induction decay of the weakly excited EQ mode.

In the region of large field enhancement (A and B), the signature of the free induction decay of the EQ mode, i.e. the persistent oscillations at 2.4 fs, is strongly enhanced in amplitude. It now approaches the amplitude of the strong scattering signal around zero. An interference dip around 3 fs indicates that both signals interfere destructively, i.e., are phase shifted by π.

These time domain field dynamics are readily understood in terms of a Fano-type interference^34^ of two (Lorentzian) oscillators.^35,36^ The EQ mode forms the spectrally narrow resonance that is interfering with a quasi-continuum formed by all other relevant Mie modes of the particle.^37,38^ This phenomenological model gives a biexponentially decaying time-domain response *r*(*t*) = Θ(*t*)*∑*_*n*=1_^2^*A*_*n*_ sin(ω_*n*_^*t*^ – *ϕ*_*n*_)exp(*−γ*_*n*_^*t*^). A simulation of the response function is shown in Section 6 of the Supporting Information. In the spectral domain, this gives the characteristic response of a Fano resonance σ(ω) = *∑*_*n*=1_^2^(*A*_*n*_/2)(*−e*^*iϕ*_*n*_^/(*ω – ω*_*n*_ + *iγ*_*n*_) + *e*^*-iϕ*_*n*_^/(ω + ω_*n*_ + *iγ*_*n*_)).^36^ Here, ω_*n*_, γ_*n*_ = 1/T2_*n*_ and *A*_*n*_*e*^*iϕ*_*n*_^ denote the energy, dephasing rate and complex amplitude of each resonance. Such a phenomenological Fano resonance model quantitatively reproduces amplitude and phase of the spectral near-field response (solid lines at A and B in Figure 3) if we assume a phase shift of ∼π between EQ and continuum at the resonance frequency of the EQ mode ω_*EQ*_.

Also, the characteristic phase jump of 2π is reproduced by this phenomenological model. Its origin is readily understood in a complex-plane phasor representation of the frequency-dependent field vectors, schematically depicted in Figure 4a. In such a diagram, the EQ resonance is represented by a circle that starts and ends at the origin in the limit of low- and high-frequency excitation, respectively. The continuum contribution is basically independent of frequency and shifts the center of the circle away from the origin. A 2π phase jump arises in σ(ω) if the shifted circle encloses the origin. This is illustrated in Figure 4a by assuming destructive interference between EQ and background at the resonance frequency ω_*EQ*_. The corresponding spectral amplitude and phase is depicted in Figure 4b-c.

**Figure 4:**
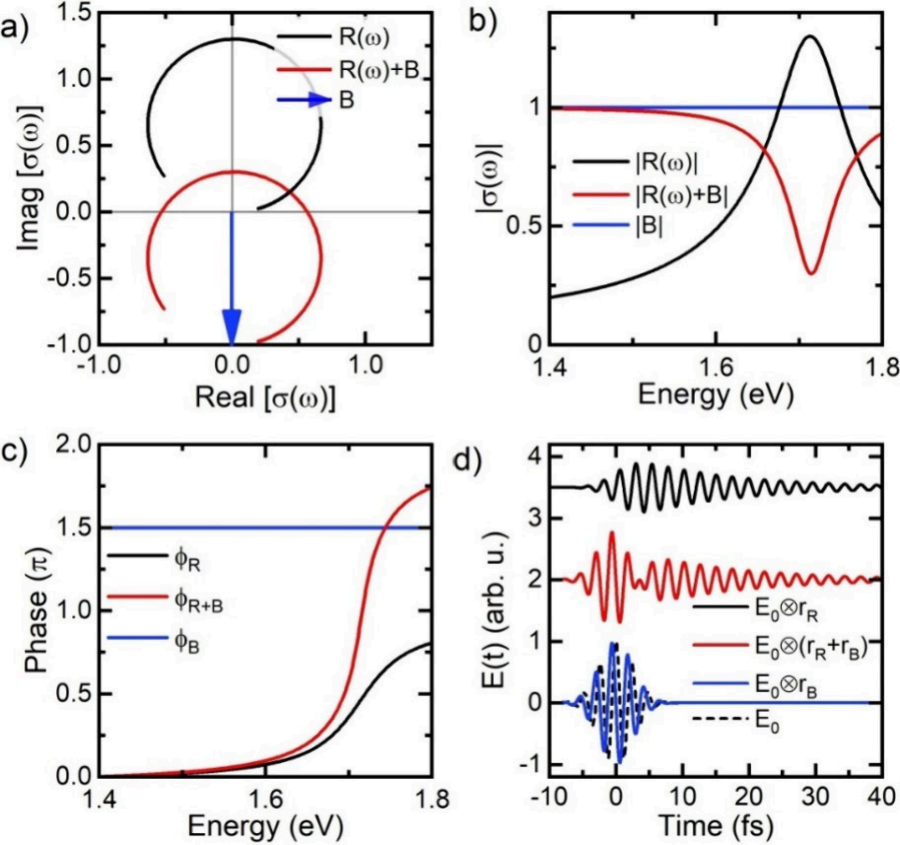
Phenomenological Lorentzian oscillator model for the response function σ(ω) for a Fano-type resonance. (a) Complex plane representation of the response function of a Lorentzian resonance *R*(ω) (black), a quasi-continuum *B*(ω) = |*B*|exp(*i*3π/2) (blue), and the resulting Fano-type interference *R*(ω)+*B*(ω) (red). (b,c) Amplitude (b) and spectral phase (c) of *R*(ω) (black), *B*(ω) (blue) and Fano resonance *R*(ω) + *B*(ω) (red). The Fano resonance exhibits a 2π phase jump if Lorentzian and background interfere destructively and the on-resonance amplitude of *R*(ω) exceeds the background amplitude. (d) Resulting time-domain near-field signal *E*(*t*) = *E*_0_(*t*)⊗*r*(*t*), for excitation with a 6 fs Gaussian-shape pulse *E*_0_ (dashed black). The convolutions with the response of the Lorentzian resonance *r*_*R*_, the background *r*_*B*_ and the Fano interference (*r*_*R*_ + *r*_*B*_) are depicted in black, blue and red, respectively.

Thus, we can conclude that the amplitude of the resonant contribution to σ_*NF*_(ω) exceeds that of the background and that the two contributions are phase shifted by ∼π at ω_*EQ*_. Importantly, this information cannot be unambiguously deduced from a measurement of |σ_*NF*_(ω)*|* alone since the same amplitude response can also be obtained if the amplitude of the resonance is smaller than that of the background. The effect of the π phase shift, between the continuum contribution and the EQ mode, on the near-field response function is illustrated schematically in Section 6 of the Supporting Information. The background field decays rapidly, within ∼2 fs, while the free-induced decay of the EQ mode persists for ∼20 fs. After convolution with *E*_0_(*t*), this gives the near-field signal shown as a red line in Figure 4d. The destructive between EQ mode and background leads to an interference dip that occurs a few femtoseconds after optical excitation. This is the characteristic sign of the Fano resonance with a 2π phase jump in the time domain.

We use Mie scattering theory for a spherical particle to rationalize this unexpected phase shift. Scattering efficiencies of the relevant modes are depicted in Figure 5a for a MAPbI_3_ particle with 250 nm radius. The narrow MQ and EQ resonances are centered at 1.41 and 1.57 eV, respectively, while the broad dipole modes are shifted to lower energies. For all modes except EQ, the spatial profile of the z-component of the scattered near-field, *E*(*s*,*z*), shows a dipolar pattern (Figure S5). Only EQ shows the additional outer ring and the field node at the rim that is seen in Figure 2b,d. Amplitude |*E*_*s*,*z*_| and spectral phase ϕ_s,*z*_ of the near-field generated by the EQ mode are depicted in Figure 5b-c as a function of the excitation energy and along a line along the polarization direction of the incident field across the surface of the nanoparticle. At the center of the particle, ϕ_*s*,*z*_ shows the characteristic π phase jump of a Lorentzian resonance. When moving toward the rim, the spectral phase ϕ_*s*,*z*_ experiences a gradual, spectrally almost constant phase shift by up to π (see cross sections at selected *x*-positions in Figure 5d-e). It is this spectrally constant phase shift between the EQ resonance and the background continuum formed by the remaining Mie modes that causes the characteristic phase jump of 2π that is uncovered at positions A and B in Figure 3.

**Figure 5:**
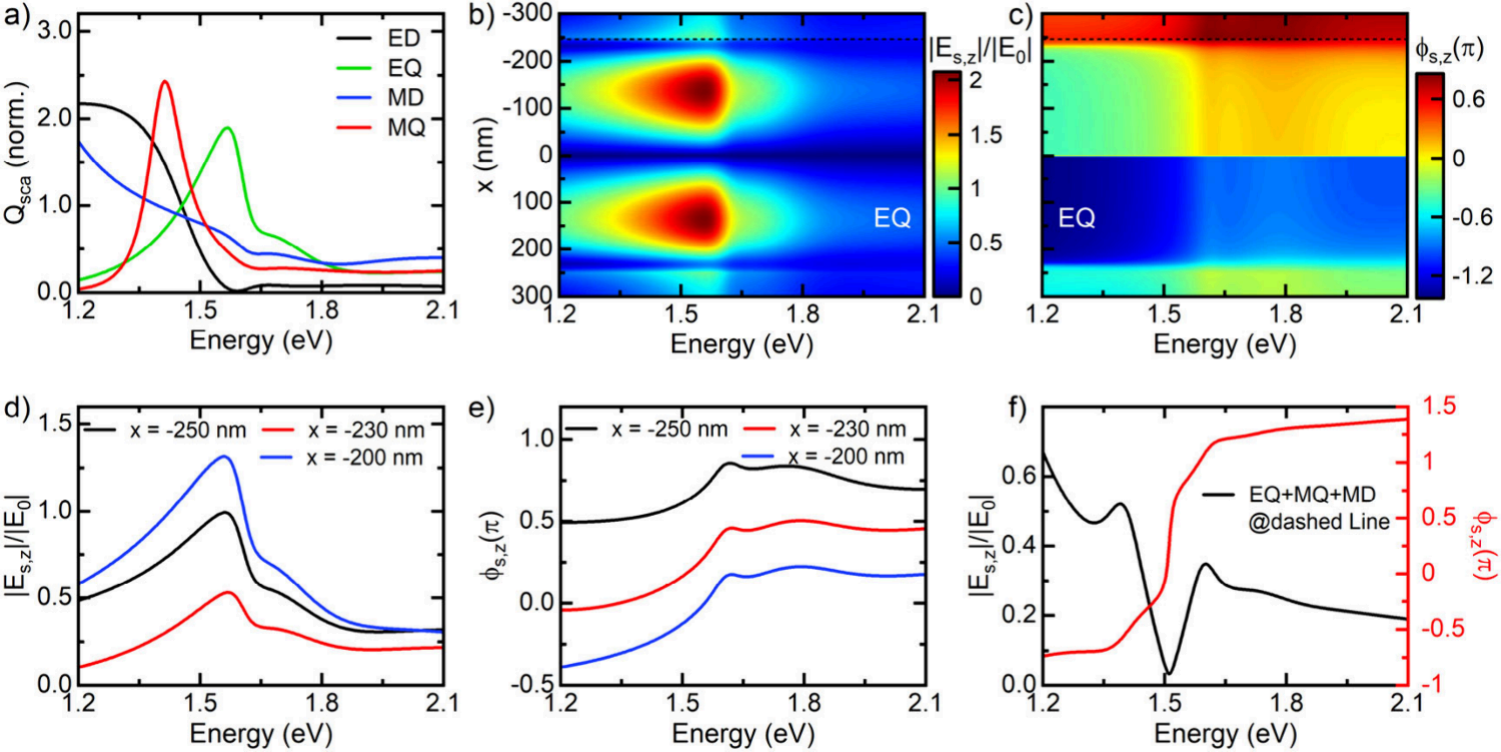
(a) Multipole decomposition of the far-field Mie scattering efficiency of a MAPbI_3_ nanosphere with 250 nm radius, optically excited with linearly x-polarized light incident along the *z*-direction. The blue (red) line depicts the magnetic dipole (quadrupole) mode (MD,MQ); the black (green) line shows the electric dipole (quadrupole) mode (ED,EQ). (b,c) Amplitude and spectral phase of the z-component of the electric near-field generated by the EQ mode at a distance of 1 nm from the surface of a MAPbI_3_ nanosphere as a function of energy and horizontal position x. The plot is taken across a line with y = 0, oriented along the incident field direction. (d,e) Amplitude phase of the EQ-mode near-field spectra at different positions close to the rim of the nanoparticle. (f) Amplitude and spectral phase of the z-component of the electric near-field, generated by the interference of the EQ, MD and MQ modes close to the rim of the nanosphere. A 2π-phase jump is observed at 1.51 eV as a result of the destructive interference of EQ and MD mode. The enhanced z-component of the electric field at 1.39 eV is the contribution of the MQ mode.

Indeed, the local near-field close to the rim of the particle that (Figure 5f) qualitatively reproduces the spectral dip and 2π phase jump at the EQ resonance. Since the experimental particles have more complex geometric shape, the phase profile depicted in Figure 5c is not expected to quantitatively match the one seen experimentally. Yet, it rationalizes the origin of observed 2π phase jump. A closer inspection of the phase profiles in Figure 5e shows that the magnitude of the phase jump at the EQ resonance decreases to less than 0.5π when moving toward the rim of the particle. Our results suggest that this is induced by the coupling of excitons and electron-pairs of the particle to the dielectric Mie resonances, i.e., it is a consequence of the hybrid Fano resonances that are formed in these particles.^17^

In summary, we have studied coherent near-field light scattering from single MAPbI_3_ nanoparticles. By combining spectral interferometry with scattering-type scanning near-field optical microscopy, we record amplitude and spectral phase of the locally scattered light in a broad spectral region around the bandgap of the particles. A direct Fourier transform of these response functions gives the time dynamics of the local optical near-field with nanometer spatial resolution. We observe signatures of hybrid Fano resonances in the light scattering from the MAPbI_3_ particles. The spectral response of the dominant electric quadrupole (EQ) mode, significantly affected by the coupling of interband excitations of the MAPbI_3_ to the shape resonances of the particle, displays a phase jump of 2π, manifesting itself as a pronounced destructive interference dip in the free induction decay of the particle a few femtoseconds after optical excitation. The observations are rationalized as destructive interference between the spectrally narrow EQ mode and the background continuum of other Mie modes coupled to interband resonances. Our results thus uncover effects of the coupling between exciton and free-carrier excitations of a semiconductor and shape resonances of a dielectric particle on the local near-field dynamics of nanoparticle. Variation of both the geometric shape and the interband absorption of such semiconducting particles can be used to tailor their optical near-field, making such particles interesting for applications in metasurfaces,^39^ topological photonics^40^ and lasing. More generally, the demonstrated spectral interferometry SNOM technique offers, with further improvements in signal-to-noise ratio and data acquisition, an interesting novel approach for probing the local optical near-field dynamics at the surface of metallic and dielectric nanoparticles, a longstanding goal in nanophotonics.

## Supporting Information Available

Sample preparation, refractive index of MAPbI_3_. experimental setup, spectral interferometry scanning near-field optical microscopy (SI-SNOM), including spectral response function and demodulation of the near-field response in SI-SNOM, data analysis, and Mie theory analysis (PDF)
