# Supplementary material for: Interferometric Near-field Fano Spectroscopy of Single Halide Perovskite Nanoparticles
Source: Nano Lett. 2024 Nov 28;24(49):15738–44. doi: 10.1021/acs.nanolett.4c04491 (PMC11638946; doi:10.1021/acs.nanolett.4c04491)
Supplement: Supplementary file 1 — nl4c04491_si_001.pdf [file nl4c04491_si_001.pdf]

Interferometric Near-field Fano Spectroscopy of Single Halide Perovskite Nanoparticles

Jinxin Zhan[1§#], Tom Jehle[1§], Sven Stephan[1], Ekaterina Tiguntseva[2], Sam S. Nochowitz[1], Petra Groß[1], Juanmei Duan[1], Sergey Makarov[2$], Christoph Lienau[1,]*

[1]Institut für Physik, Carl von Ossietzky Universität, 26129 Oldenburg, Germany.

[2]Department of Nanophotonics and Metamaterials, ITMO University, St. Petersburg, 197101, Russia

[*]Correspondence to: christoph.lienau@uni-oldenburg.de

1. Sample preparation

Perovskite nanoparticles are fabricated by a laser printing method from a perovskite thin film.[1, 2] For preparing a uniform and dense perovskite film, a solution of perovskite precursor ($MAPbI_3$) is firstly prepared in a drybox as follows: methylammonium iodide (MAI) in γ-butyrolactone with dimethyl sulfoxide (GBL/DMSO) at the concentration of 1.5 M is used to dissolve 1.5 M of lead iodine (PbI_2). The solution is stirred and heated at 70 °C overnight and used after filtration through 0.45 μm PTFE syringe filter. With this solution, a perovskite layer is created by a solvent engineering technique inside the drybox.[3] First, the solution precursor $MAPbI_3$ is spread over the entire substrate surface at rotation speed 1000 rpm. The solvent is evaporated after maintaining the rotation for several tens of seconds. Then, 200 μL of toluene is dripped at 3000 rpm. During film formation, the toluene does not dissolve perovskite. Finally, the formed film is annealed at 100 °C for 10 min, converting the complex into highly uniform and crystalline perovskite.

For the laser printing method, we employ a forward-transfer geometry. The receiving glass substrate is washed by sonication in deionized water, toluene, acetone, and isopropanol. Yb^{3+} femtosecond laser pulses at λ = 1050 nm with energy around 50 nJ are tightly focused by a 10× objective with numerical aperture (NA) of 0.26 to a spot size of approximately 5 μm. A perovskite film is placed on a three-dimensional air-bearing translating stage (ABL1000, Aerotech), allowing translation of the sample with an accuracy of better than 100 nm. The nanoparticles were fabricated from a smooth surface in a single-shot regime in the forward-transfer (LIFT) geometry when the receiving substrate was placed under the film with a spacing of ∼50 μm. The morphology and size of the nanoparticles has been studied using scanning electron microscopy (SEM) with an electron microscope. Characteristic images are shown in the Supporting Information of Ref. 2.

2. Refractive index of $MAPbI_3$

The frequency dependence of the complex-valued refractive index $\tilde{n} = n + ik$ of bulk $MAPbI_3$ that has been used for all calculations is shown in Fig. S1.[4] Interband absorption sets in around 755 nm. Excitonic contributions to the refractive index are weak.

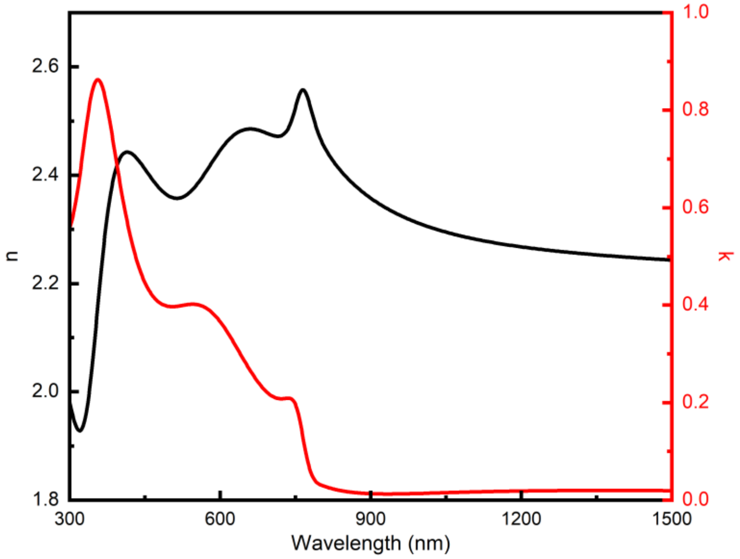

Figure S1. Wavelength-dependent refractive index (n) and extinction coefficient (k) for perovskite $MAPbI_3$.

3. Experimental setup

The experimental setup is schematically depicted in Fig. 1 of the main manuscript. All SI-SNOM experiments are performed using a home-built tapping mode atomic force microscope (AFM).[5-7] A chemically etched gold taper[8] with a radius of curvature of 20 nm is used as a near-field probe. The tuning fork is electrically driven to periodically modulate the tip-sample distance at frequency ω_m with an amplitude of 12 nm and probes the tip-sample interaction. A feedback loop is used to maintain a minimum tip-sample distance of 3 nm. The sample is scanned relative to the tip using a three-axis nanopositioner (Physik Instrumente, PI-733.3DD).

For the optical experiments, we use light from a 6-fs Titanium-sapphire laser operating at a repetition rate of 80 MHz. The spectrum of the laser covers the range from 670 nm to 900 nm. The laser is sent into a Michelson interferometer and split into a sample and reference beam using a broad band beam splitter (BS). The beam in the sample arm with a power of a few mW is focused onto the tip apex using a reflective microscope objective (MO, Beck Optronics Solutions, model 5003-000) with a numerical aperture of 0.4. The light that is scattered from the tip-sample junction is collected using the same objective in a back-reflection geometry. On BS, it is overlapped with the time-delayed beam in the reference arm. Two detection systems are used. An APD (Hamamatsu C12702-03) that is connected to a lock-in amplifier (Zurich Instruments HFLI) detects the intensity of the tip-scattered light in the absence of a reference beam. This signal is demodulated at different harmonics of ω_m. The signal at the third harmonic is used to record a spatial map of the optical near-field distribution by raster-scanning the sample relative to the fixed tip position using the 3-axis nanopositioner. Exemplary results of these measurements are shown in Fig. 2b of the main manuscript.

Spectral interferograms (SI) of the signal scattered from the tip apex and the reference laser are measured using a home-built detection setup. For this, signal and reference beam are spectrally dispersed in a low-astigmatism monochromator (Princeton Instruments, IsoPlane-160) and then recorded with a fast line scan camera (e2V AViiVA EM4 with 512 pixels) operating at a read-out frequency of 210 kHz. This allows us to measure individual SI with an acquisition time of 4.6 µs. This acquisition time is shorter than the tip oscillation period of ~40 µs and thus enables us to record spectra at different tip-sample distances. Also, the short acquisition time suppresses any mechanical motion of the interferometer and therefore ensures phase stability of the SI.

Experimentally, we record up to 60000 spectra at each sample position during a total acquisition time of 0.28 seconds.

4. Spectral interferometry scanning near-field optical microscopy (SI-SNOM): Spectral response function

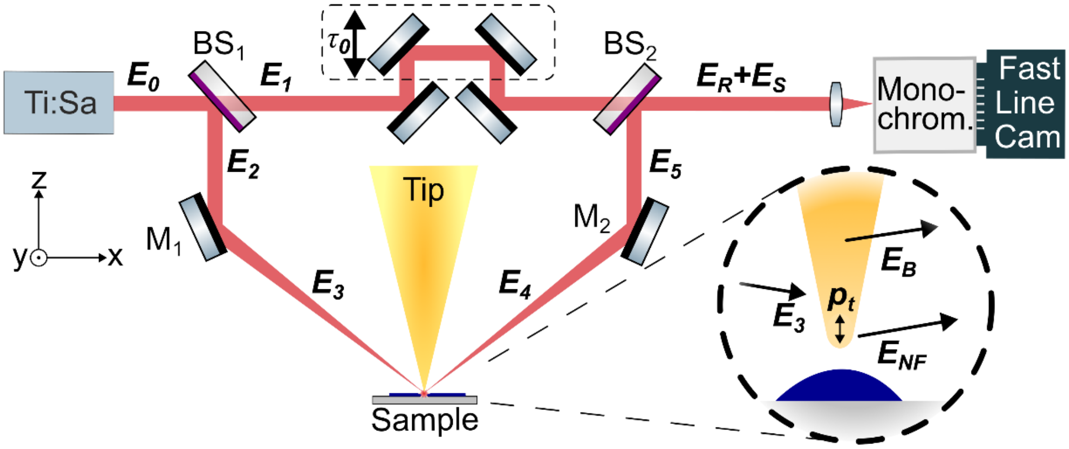

Figure S2: Schematic description of Spectral Interferometry Scanning Near-field Optical Microscopy (SI-SNOM). Light from a broadband femtosecond laser is split into a reference and signal arm at beam splitter BS1. The signal beam is focused onto the scattering type near field probe (tip). The dashed insert shows a magnification of the tip-sample junction. Both optical near field scattering from the tip-sample junction and background light scattered from the tip shaft is collected and overlapped with the reference pulses, time-delayed by a variable delay τ_0, on BS2. Both signal and reference pulses are sent through a monochromator. SIs of the sample and reference pulses are recorded at a repetition rate of 210 kHz using a fast line scan camera.

The aim of this section is to introduce the spectral response function σ that connects the signal field $E_s = \sigma E_R$ and the reference field E_R in a broadband Spectral Interferometry Scanning Near-field Optical Microscope (SI-SNOM), as schematically depicted in Fig. S2. For this, we discuss the propagation of a monochromatic incident plane wave $\boldsymbol{E_0}$ with angular frequency ω and

wavevector $\boldsymbol{k_0} = k_0\boldsymbol{n_0} = k_0\boldsymbol{n_x}$ (unit vectors $\boldsymbol{n_0} = \boldsymbol{n_x}$) along the x-axis through the SI-SNOM interferometer:

$$\boldsymbol{E_0}(\boldsymbol{r}, t) = E_0\boldsymbol{n_z}e^{i\boldsymbol{k_0}\boldsymbol{r}}e^{-i\omega t} \tag{1}$$

The magnitude of the wave vector is given by $k_0 = \frac{\omega}{c}$. We assume linear polarization along the z-direction $\boldsymbol{n_z}$. A similar discussion holds for y-polarized light. The plane wave impinges onto a 50/50 beam splitter (BS_1) with complex, frequency-dependent transmission t_{BS1} and reflectivity r_{BS1}. It is split into a beam $\boldsymbol{E_1} = t_{BS1}\boldsymbol{E_0}$ in the reference arm and a beam in the signal arm. The beam $\boldsymbol{E_1}$ passes through a variable time delay τ_0 and a second beam splitter (BS_2) to give the reference field

$$\boldsymbol{E_R} = t_R e^{i\omega\tau_0}\boldsymbol{E_0} = E_R\boldsymbol{n_z}e^{i\omega\tau_0} \tag{2}$$

where $t_R = t_{BS2}r_{RM}t_{BS1}$is a combined transmission constant and r_{RM} accounts for the reflectivity of the mirrors from the delay stage. The reference field points in the same direction as the incident beam.

The field in the signal arm

$$\boldsymbol{E_2} = \overleftrightarrow{\boldsymbol{M}}_{\boldsymbol{20}} r_{BS1}\boldsymbol{E_0} \tag{3}$$

propagates along $\boldsymbol{k_2} = k_0\boldsymbol{n_2}$, and its field vector points along $\overleftrightarrow{\boldsymbol{M}}_{\boldsymbol{20}}\boldsymbol{n_z}$. The matrix $\overleftrightarrow{\boldsymbol{M}}_{\boldsymbol{ij}} = \boldsymbol{n_i}\boldsymbol{n_j}$ describes the rotation of the wave vector from $\boldsymbol{n_j}$ to $\boldsymbol{n_i}$ upon reflection off the mirror. It is given by the dyadic (outer) product $\boldsymbol{n_i}\boldsymbol{n_j}$ of the wave vectors.[9]

A mirror M1 steers the signal beam onto the apex of the near field probe, the tip of a sharp gold taper. The linearly polarized field at the tip apex propagates along $\boldsymbol{k_3} = k_0\boldsymbol{n_3}$ and can approximately be written as:

$$\boldsymbol{E_3} = \overleftrightarrow{\boldsymbol{M}}_{\mathbf{32}} r_{M1} \boldsymbol{E_2} \tag{4}$$

where r_{M1} is the reflection coefficient of the mirror. In practice, the signal beam is focused onto the tip apex using a microscope objective, maintaining the linear polarization of the incident beam. Hence r_{M1} should, more generally, be understood as an effective transmission coefficient of the entire focusing optics in the experimental setup.

The field $\boldsymbol{E_3}$ couples to the tip-sample junction and induces an effective dipole moment $\boldsymbol{p_t}(d) = \overleftrightarrow{\boldsymbol{\alpha}}_{\boldsymbol{eff}}(\omega, d)\boldsymbol{E_3}$ at the tip apex. Here, $\overleftrightarrow{\boldsymbol{\alpha}}_{\boldsymbol{eff}}(\omega, d)$ represents the effective polarizability tensor of the coupled tip-sample system.[10-12] This effective polarizability sensitively probes the near-field coupling between tip and sample and therefore depends sensitively on their distance d.[5, 12] In case that the coupling is sufficiently weak, first order perturbation theory holds and the spectral dependence of $\overleftrightarrow{\boldsymbol{\alpha}}_{\boldsymbol{eff}}$ is distance independent, $\overleftrightarrow{\boldsymbol{\alpha}}_{\boldsymbol{eff}} = \overleftrightarrow{\boldsymbol{\alpha}}_{\omega}(\omega)\boldsymbol{\alpha_d}(d)$.[5] For stronger couplings, this separation can no longer be made and the distance dependence of the spectral shape of $\overleftrightarrow{\boldsymbol{\alpha}}_{\boldsymbol{eff}}$ becomes important.[5] This tip dipole then emits a field $\boldsymbol{E_{NF}} = \frac{\omega^2}{\epsilon_0 c^2}\overleftrightarrow{\boldsymbol{G}}_{\mathbf{0}}\overleftrightarrow{\boldsymbol{\alpha}}_{\boldsymbol{eff}}\boldsymbol{E_3}$ into the far field, where $\overleftrightarrow{\boldsymbol{G}}_{\mathbf{0}}$ is the far-field dyadic Greens tensor.[13]

In addition to this desired "near-field" emitted from the tip dipole, a fraction of the incident field $\boldsymbol{E_3}$ is also scattered from other sources near the tip apex and the sample. This results in an additional undesired background field $\boldsymbol{E_B}$ with much weaker dependence on the tip-sample distance than $\boldsymbol{E_{NF}}$. Quite generally, $\boldsymbol{E_B}$ can be expressed as $\boldsymbol{E_B} = \overleftrightarrow{\boldsymbol{M}}_{\boldsymbol{B'}}\boldsymbol{E_3}$ with $\overleftrightarrow{\boldsymbol{M}}_{\boldsymbol{B'}}$ denoting a scattering tensor for the background field. Both scattered near-field and background field are collected and collimated using a microscope objective, giving the collected field $\boldsymbol{E_4} = \boldsymbol{E_{NF}} + \boldsymbol{E_B}$ that is propagating in direction $\boldsymbol{n_4}$. This beam is then sent onto BS_2 using M_2, creating $\boldsymbol{E_5} = \overleftrightarrow{\boldsymbol{M}}_{\mathbf{54}} r_{M2} \boldsymbol{E_4}$.

After BS_2 the signal beam $\boldsymbol{E_S} = r_{BS2}\overleftrightarrow{\boldsymbol{M}}_{\boldsymbol{65}}\boldsymbol{E_5}$ is spatially overlapped with the reference beam. We can rewrite $\boldsymbol{E_S} = \left(\overleftrightarrow{\boldsymbol{M}}_{\boldsymbol{NF}} + \overleftrightarrow{\boldsymbol{M}}_{\boldsymbol{B}}\right) \cdot \boldsymbol{E_0}$ with

$$\overleftrightarrow{\boldsymbol{M}}_{\boldsymbol{NF}}(d) = t_{NF}\overleftrightarrow{\boldsymbol{M}}_{\boldsymbol{64}}\overleftrightarrow{\boldsymbol{G}}_{\boldsymbol{0}}\overleftrightarrow{\boldsymbol{\alpha}}_{\boldsymbol{eff}}(d)\overleftrightarrow{\boldsymbol{M}}_{\boldsymbol{30}} \tag{5}$$

and

$$\overleftrightarrow{\boldsymbol{M}}_{\boldsymbol{B}} = t_B\overleftrightarrow{\boldsymbol{M}}_{\boldsymbol{64}}\,\overleftrightarrow{\boldsymbol{M}}_{\boldsymbol{B'}}\overleftrightarrow{\boldsymbol{M}}_{\boldsymbol{30}} \tag{6}$$

Here, $t_{NF} = r_{BS2}r_{M2}\frac{\omega^2}{\epsilon_0 c^2}r_{M1}r_{BS1}$ and $t_B = r_{BS2}r_{M2}r_{M1}r_{BS1}$. The transfer matrix $\overleftrightarrow{\boldsymbol{M}}_{\boldsymbol{NF}}(d)$ has a strong distance dependence resulting from change in the effective tip polarizability when approaching the surface. Both signal and reference beams are sent through a monochromator and the spectral interferogram (SI)[14, 15]

$$S(\omega) = |\boldsymbol{E_R}(\omega,\tau_0) + \boldsymbol{E_S}(\omega,d)|^2 \tag{7}$$

is recorded using a fast line scan camera. Importantly, the integration time of the camera of 4.6 µs is sufficiently short to suppress any undesired mechanical oscillations of the interferometer during the acquisition time of one spectrum. Also, it is much shorter than the modulation period of the tip-sample distance. Hence, the tip-sample distance can be considered as being constant during the recording of one spectrum. This is the key to recording distance-dependent SI and, thus, to subtract the contribution of the undesired background field from the SI.

The measured SI can be expanded as

$$S(\omega) = I_R(\omega) + I_S(\omega) + E_R(\omega)E_S^*(\omega)e^{i\omega\tau_0} + E_R^*(\omega)E_S(\omega)e^{-i\omega\tau_0} \tag{8}$$

with $I_R(\omega) = |\boldsymbol{E_R}(\omega)|^2$, $I_s(\omega) = |\boldsymbol{E}_s(\omega)|^2$and $E_s(\omega) = \boldsymbol{E}_s(\omega) \cdot \boldsymbol{n}_z$. Now, we introduce the scalar response function $\sigma(\omega, d)$ as

$$E_S(\omega, d) = \sigma(\omega, d)E_R(\omega) \tag{9}$$

to rewrite the spectral interferogram as

$$S(\omega) = I_R(\omega) + I_S(\omega) + I_R(\omega)\left(\sigma^*(\omega, d)e^{i\omega\tau_0} + \sigma(\omega, d)e^{-i\omega\tau_0}\right). \tag{10}$$

This response function can be separated into the response functions for the near-field and the background signals $\sigma(\omega, d) = \sigma_{\mathrm{NF}}(\omega, d) + \sigma_{\mathrm{B}}(\omega)$, which can be written as

$$\sigma_{NF}(\omega, d) = \left(\overleftrightarrow{\boldsymbol{M}}_{\boldsymbol{NF}}(\omega, d)\right)_{\boldsymbol{zz}} / t_R, \tag{11}$$

$$\sigma_B(\omega) = \left(\overleftrightarrow{\boldsymbol{M}}_{\boldsymbol{B}}(\omega)\right)_{\boldsymbol{zz}} / t_R. \tag{12}$$

5. Demodulation of the near-field response in SI-SNOM

Since the near field response function $\sigma_{NF}(\omega, d)$ depends sensitively on the tip-sample distance, it can be isolated by temporally modulating the tip-sample distance, as is commonly done in scattering type SNOM.[10, 16] For this, we proceed as follows. The near-field probe is mounted on a quartz tuning fork that is periodically driven at its resonance frequency f of ~ 28 kHz. This modulates the tip-sample distance $d(t_m) = d_0 + \Delta d \cos(\omega_m t_m + \varphi_m)$ with an amplitude Δd of 12 nm and at $\omega_m = 2\pi f$. A feed-back loop is used to maintain a minimum tip-sample distance of 3 nm while raster-scanning the tip across the sample. At every position $\boldsymbol{r}_t$ on the sample, a series of N ($N = 5.000 - 60.000$) SIs $S_i\left(\omega, d(t_k)\right), k = 1, \ldots, N$ is recorded. The time interval Δt between adjacent spectra is 4.6 µs.

A direct Fourier transformation of each SI gives the time-domain signal $s_k(t) = \mathcal{F}(S_k(\omega)) = s_{k-}(t + \tau_{0k}) + s_{k0}(t) + s_{k+}(t - \tau_{0k})$, containing three peaks centered at $t = -\tau_{0k}, 0, \tau_{0k}$. With $\otimes$ denoting convolution and $\delta(t)$ being the Dirac delta distribution, this time-domain representation can in good approximation be expressed as

$$s_k(t) \approx I_{Rk}(t) \otimes [\delta(t) + \sigma_k(t) \otimes \sigma_k^*(t) + \sigma_k(t - \tau_{0k}) + \sigma_k^*(t + \tau_{0k})]. \tag{13}$$

$$s_{0k}(t) = I_{Rk}(t) \otimes [\delta(t) + \sigma_k(t) \otimes \sigma_k^*(t)] \tag{14}$$

$$s_{+k}(t) = I_{Rk}(t) \otimes \sigma_k(t - \tau_{0k}) \quad (15)$$

$$s_{-k}(t) = I_{Rk}(t) \otimes \sigma_k^*(t + \tau_{0k}) \quad (16)$$

This neglects possible polarization rotations of the signal field $\boldsymbol{E}_S(\omega)$ relative to the reference field $\boldsymbol{E}_R(\omega)$. For sufficiently strong reference fields such effects can safely be ignored since they enter the signal $s_k(t)$ only through the small quadratic term in Eq. 14. In the experiment, the time delays τ_{0k} are chosen to be larger than the temporal width of each peak. Therefore, the three components can be isolated. In a first step, the time delays τ_{0k} are deduced from the temporal phase of the sidepeaks s_{k-} and s_{k+}. Then, all sidepeaks are set to identical time delays $\bar{\tau}_0$

$$\bar{s}_{+k}(t) = s_{+k}(t) \exp\big(i\omega(\tau_0 - \tau_{0k})\big) \quad (17)$$

$$\bar{s}_{-k}(t) = s_{-k}(t)\, exp\big(-i\omega(\tau_0 - \tau_{0k})\big) \quad (18)$$

and time-shifted time-domain signals are calculated as

$$\bar{s}_k(t) = \bar{s}_{-k}(t + \bar{\tau}_0) + s_{0k}(t) + \bar{s}_{+k}(t - \bar{\tau}_0) \quad (19)$$

Inverse Fourier transform of the zero-delay component gives $\bar{S}_{0k}(\omega) = \mathcal{F}^{-1}(s_{0k}(t)) = I_{Rk}(\omega)(1 + |\sigma_k(\omega)|^2)$, containing mainly the information about the incident spectrum $I_{Rk}(\omega)$ as $|\sigma_k(\omega|^2 \ll 1$. Averaging over all N spectra gives $\bar{S}_0(\omega) = (1/N) \sum_k \bar{S}_{0k}(\omega)$. Inverse Fourier transform of the two sidepeaks gives $\bar{S}_{-k}(\omega) = \mathcal{F}^{-1}(\bar{s}_{-k}(t)) = I_{Rk}(\omega)\sigma_k^*(\omega)$ and $\bar{S}_{+k}(\omega) = I_{Rk}(\omega)\sigma_k(\omega)$ and, after averaging, $\bar{S}_{+/-}(\omega) = (1/N) \sum_i \bar{S}_{+/-,k}(\omega)$. Normalization $\bar{S}_0(\omega)/\bar{S}_+(\omega)$ cancels out the incident spectrum and forms a quadratic equation for the magnitude of the average response function $|\bar{\sigma}(\omega)| = (1/N) \sum_k |\sigma_k(\omega)|$: $\bar{S}_0/|\bar{S}_+| = (1+|\bar{\sigma}(\omega)|^2)/|\bar{\sigma}(\omega)|$.

Solving this equation for $|\bar{\sigma}(\omega)| < 1$ gives

$$|\bar{\sigma}(\omega)| = \frac{\bar{S}_0(\omega)}{2|\bar{S}_+(\omega)|} - \sqrt{\frac{\bar{S}_0(\omega)^2}{4|\bar{S}_+(\omega)|^2} - 1} \quad (20)$$

We only use this quantity to extract the averaged reference spectrum

$\bar{I}_R(\omega) = \bar{S}_0(\omega)/(1 + |\bar{\sigma}(\omega)|^2)$.

With this, the complex-valued response function $\bar{\sigma}(\omega)$ can be deduced as $\bar{\sigma}(\omega) = \bar{S}_+(\omega)/\bar{I}_R(\omega)$. This response function still contains contribution from near-field and background scattering and thus does not allow us yet to deduce the desired near-field response function $\sigma_{NF}(\omega, d)$.

For this, we calculate normalized spectral response functions as $\bar{\sigma}_{+/-,k}(\omega) = \bar{S}_{+/-,k}(\omega)/\bar{I}_R(\omega)$. We then demodulate these response functions at the n-th harmonic of the tip modulation frequency

$$\sigma_+^{(n)}(\omega) = \Delta t \sum_k \bar{\sigma}_{+k}(\omega)\, cos(kn\omega_m \Delta t) \quad (21)$$

$$\sigma_-^{(n)}(\omega) = \Delta t \sum_k \bar{\sigma}_{-k}^*(\omega)\, cos(kn\omega_m \Delta t) \quad (22)$$

The time axis is chosen such that time zero corresponds to a point of nearest distance between tip and sample. The demodulation integral effectively forms weighted averages over the distance dependence of the response function. Since the background scattering signals vary on a scale of half the wavelength, their contributions to the response functions with $n \geq 2$ are small and will be neglected in the following.[16, 17] For $n \geq 2$, we identify the n-th order near-field response function as

$$\sigma_{NF,+/-}^{(n)}(\omega) = \sigma_{+/-}^{(n)}(\omega), \qquad n \geq 2 \quad (23)$$

Experimentally we find that the 2nd order response functions show negligible background contributions. We therefore use this order to deduce the desired complex-valued near-field response function

$$\sigma_{NF}(\omega) = \sigma_{+}^{(2)}(\omega) \tag{24}$$

The response functions are recorded at every tip sample position $\boldsymbol{r}_t$. To correct for the contributions of the finite time delay in the reference arm and possible chirp in the setup, we perform a reference measurement of the spectral phase of the response at a sample position $\boldsymbol{r}_{ref}$ on the substrate area of the sample, with negligible resonant light scattering from the $MaPbI_3$ particles. The analysis of this measurement gives the reference phase as $\varphi_{ref}(\omega) = \mathrm{Arg}(\sigma_{NF}(\omega, \boldsymbol{r}_{ref})$. Alternatively, also the zero-order demodulated response function at the sample position, $\sigma_{+}^{(0)}(\omega, \boldsymbol{r}_t)$, may be used for referencing, since it is largely dominated by background scattering, $\varphi_{ref}(\omega) = \mathrm{Arg}(\sigma_{+}^{(0)}(\omega, \boldsymbol{r}_t)$. The spectral phase of the near-field response function at position $\boldsymbol{r}_t$ is then taken as $\varphi_{NF}(\omega, \boldsymbol{r}_t) = \mathrm{Arg}\big(\sigma_{NF}(\omega, \boldsymbol{r}_t)\big) - \varphi_{ref}(\omega)$. A direct Fourier transform gives the real-valued time-domain near-field response function $r_{NF}(t, \boldsymbol{r}_t)$ at position $\boldsymbol{r}_t$ as

$$r_{NF}(t, \boldsymbol{r}_t) = Re\big(\mathcal{F}(\sigma_{NF}(\omega, \boldsymbol{r}_t)\big) = \frac{1}{2\pi} Re \int \sigma_{NF}(\omega, \boldsymbol{r}_t)\, e^{i\varphi_{NF}(\omega, r_t)} e^{-i\omega t} d\omega. \tag{25}$$

6. Data analysis

In the following, we illustrate the analysis of the SI. For this, we consider the SI recorded at position A in Fig. 3 of the main text. The data are taken at a point on the surface of the $MAPbI_3$ particle with large near field enhancement. Fig. S3a shows the time series of SIs recorded with the fast line camera. All SIs are set to the same time delay $\bar{\tau}_0$ using Eqs (17) and (18). The data demonstrate the excellent signal to noise ratio of the spectra recorded with the fast line scan camera. The amplitude of the reference laser spectrum $\bar{I}_R(\omega)$ is depicted as a black line in Fig. S3b. It is deduced from an analysis of the central peak of the time-domain signals $s_k(t)$, as discussed in Section 2. The signal closely matches the spectrum of the excitation laser.

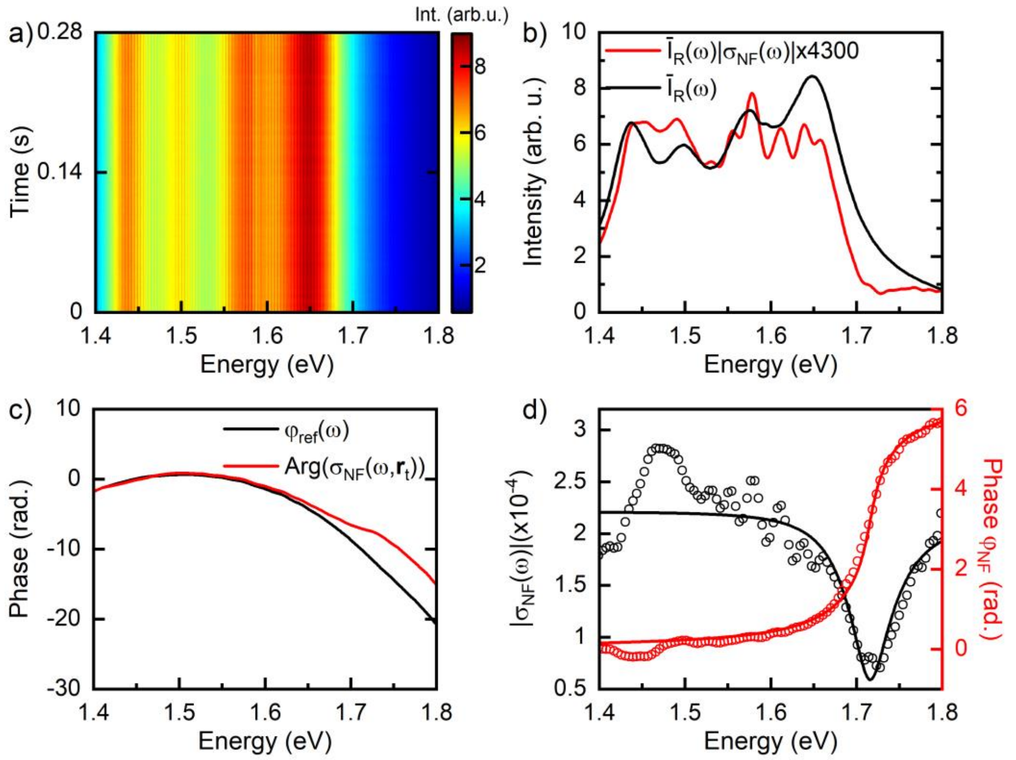

Figure S3. SI analysis. a) Time series of 60.000 SIs recorded at a fixed position (position A in Fig. 4) with large near field enhancement on the surface of the $MAPbI_3$ particle. All SIs are set to a fixed time delay $\bar{\tau}_0$ using Eqs. (17) and (18). b) Amplitude of the reference beam $\bar{I}_R(\omega)$ (black line) together with the amplitude of the near-field signal $\bar{I}_R(\omega)|\sigma_{NF}(\omega)|$ scattered from the tip-sample junction (red line). c) Spectral phase $\mathrm{Arg}(\sigma_{NF}(\omega,\boldsymbol{r}_t))$ of the near-field signal (red line) together with the reference phase $\varphi_{ref}(\omega)$ taken from the zero-order demodulated signal (black line). d) Amplitude $|\sigma_{NF}(\omega)|$ (black circles) and spectral phase $\varphi_{NF}(\omega,\boldsymbol{r}_t)$ (red circles) of the near-field response function. The characteristic dip and phase jump around 1.72 eV is the signature of the Fano resonance at the position of the electric quadrupole mode of the particle. Solid lines are fits to a Fano-resonance line shape model (explained in the main text).

The red line in Fig. S3b displays the amplitude of the spectrum $\bar{I}_R(\omega)|\sigma_{NF}(\omega)|$ that is deduced from $\sigma_+^{(2)}(\omega)$, Eq. (21). The ratio of this spectrum and the reference spectrum gives the amplitude of the spectral near-field response $|\sigma_{NF}(\omega)|$ that is shown in Fig. S3d as black open circles. Fig. S3c shows, as a black line, the spectral phase reference $\varphi_{ref}(\omega) = \mathrm{Arg}\left(\sigma_+^{(0)}(\omega,\boldsymbol{r}_t)\right)$ that is taken

from the zero-order demodulated signal. An approximately quadratic phase dependence reflects the residual chirp in the reference and signal arms of the interferometer. The spectral phase $\mathrm{Arg}\big(\sigma_{NF}(\omega, \boldsymbol{r}_t)\big)$ of the near-field signal that is scattered from the tip-sample junction is depicted as a red line in Fig. S3c. It shows, in addition to the quadratic phase dependence, a characteristic phase jump by 2π at a resonance energy of 1.7 eV. The difference between the two curves gives the spectral phase of the near-field response $\varphi_{NF}(\omega, \boldsymbol{r}_t)$ that is depicted in Fig. S3d as red open circles. The solid lines in Fig. S3d are fits to the Fano-resonance line shape model explained in the main text. Figure S4b shows the corresponding biexponential time domain response function $r_{NF}(t)$ obtained by direct Fourier transform of the fitted response function $\sigma_{NF}(\omega)$, the local near field dynamics are given by convolution with the bandwidth-limited electric field profile $E_0(t)$ of the excitation laser are depicted in Fig. S4c.

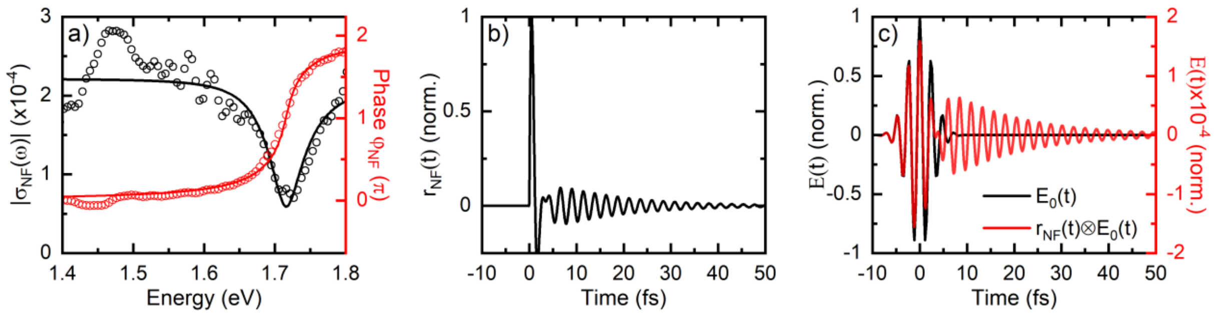

Fig. S4**:** (a) Amplitude (black circles) and spectral phase (red circles) of the local near-field response function $\sigma_{NF}(\omega)$ recorded at the rim of a $MAPbI_3$ particle, at position A in Fig. 3. The data show an absorption dip at 1.72 eV, together with a phase jump of 2π around this energy. This is the signature of a Fano resonance which can be explained by the interference of a spectrally narrow and a spectrally broad Lorentzian resonance. Amplitude and spectral phase of the response function that arises from this interference of two Lorentzians are shown as solid lines. (b) Time-domain response function $r_{NF}(t)$ obtained by direct Fourier transformation of the fitted response $\sigma_{NF}(\omega)$. Interference between the two resonances results in a biexponential response in the time domain. (c) Local near-field dynamics (red line) obtained by convoluting $r_{NF}(t)$ with the bandwidth-limited electric field profile $E_0(t)$ of the excitation laser (black line).

7. Mie theory analysis of the near field distributions of the $MAPbI_3$ particles

Analytical Mie theory[18] is used to estimate the optical near fields at the surface of a $MAPbI_3$ particle. We take a MATLAB-based implementation of Mie theory[19] as basis for our calculations and consider the scattering of linearly polarized plane wave by a spherical particle of radius a embedded in a homogeneous medium. The complex-valued refractive index of the sphere is n_1 and that of the embedding medium n_0. We assume that the incident wave with amplitude E_0 is polarized along x and propagates in z-direction. The relevant formulas for calculating optical near fields are summarized in the following for completeness.

We start by calculating the Mie coefficients a_n and b_n:

$$a_n = \frac{m\psi_n(mx)\psi'_n(x) - \psi_n(x)\psi'_n(mx)}{m\psi_n(mx)\xi'_n(x) - \xi_n(x)\psi'_n(mx)}, \quad (26a)$$

$$b_n = \frac{\psi_n(mx)\psi'_n(x) - m\psi_n(x)\psi'_n(mx)}{\psi_n(mx)\xi'_n(x) - m\xi_n(x)\psi'_n(mx)}. \quad (26b)$$

Here $\psi_n(\rho)$ and $\xi_n(\rho)$ are Riccati-Bessel functions defined by Bessel functions of the first kind, $J_n(\rho)$, and Bessel functions of the third kind, $H_n^{(1)}(\rho)$, in the following way:

$$\psi_n(\rho) = \sqrt{\frac{\pi\rho}{2}} J_{n+\frac{1}{2}}(\rho), \quad (27a)$$

$$\xi_n(\rho) = \sqrt{\frac{\pi\rho}{2}} H^{(1)}_{n+\frac{1}{2}}(\rho). \quad (28b)$$

Here, the size parameter $x = ka$, with k being the magnitude of the wave vector of the incident light. The relative refractive index $m = n_1/n_0$. The dimensionless variable $\rho = kr$ with r being the radial distance from the center of the sphere.

Using these Mie coefficients the total far field scattering cross section C_{sca} is directly accessible:

$$C_{sca} = \frac{2\pi}{k^2}\sum_{n=1}^{\infty}(2n+1)\left(|a_n|^2 + |b_n|^2\right), \tag{29}$$

the scattering efficiency is then given by

$$Q_{sca} = \frac{C_{sca}}{\pi a^2}. \tag{30}$$

For the particle sizes $a \leq 300\ nm$ that are examined in the present paper mostly the contributions of the dipole modes, $n = 1$, and quadrupole modes, $n = 2$, to the scattering cross section are relevant. The coefficients a_n give the amplitudes of the electric modes (transverse magnetic (TM)) with vanishing radial magnetic field component, while the b_n give the amplitudes of the magnetic modes (transverse electric (TE)) with vanishing radial electric field component. The scattered electric and magnetic near fields outside of the sphere are given as:

$$\boldsymbol{E}_s = \sum_{n=1}^{\infty} E_n(ia_n\boldsymbol{N}_{e1n} - b_n\boldsymbol{M}_{o1n}), \tag{31}$$

$$\boldsymbol{H}_s = \frac{k}{\omega\mu}\sum_{n=1}^{\infty} E_n(ib_n\boldsymbol{N}_{o1n} + a_n\boldsymbol{M}_{e1n}), \tag{32}$$

$$E_n = i^n E_0 \frac{2n+1}{n(n+1)}, \tag{33}$$

where μ is the magnetic permeability of the embedding medium. In spherical coordinates, the spherical vector harmonics can be expressed as:[18]

$$\boldsymbol{M}_{e1n} = -\sin(\phi)\pi_n(\theta)h_n^{(1)}(\rho)\hat{e}_\theta - \cos(\phi)\,\tau_n(\theta)h_n^{(1)}(\rho)\hat{e}_\phi, \tag{34a}$$

$$\boldsymbol{M}_{o1n} = \cos(\phi)\pi_n(\theta)h_n^{(1)}(\rho)\hat{e}_\theta - \sin(\phi)\,\tau_n(\theta)h_n^{(1)}(\rho)\hat{e}_\phi, \tag{34b}$$

$$\boldsymbol{N}_{e1n} = \frac{h_n^{(1)}(\rho)}{\rho}\cos(\phi)\,n(n+1)\sin(\theta)\,\pi_n(\theta)\hat{e}_r + \cos(\phi)\,\tau_n(\theta)\frac{1}{\rho}\frac{\partial}{\partial\rho}\left[\rho h_n^{(1)}(\rho)\right]\hat{e}_\theta \tag{34c}$$

$$-\sin(\phi)\,\pi_n(\theta)\frac{1}{\rho}\frac{\partial}{\partial\rho}\left[\rho h_n^{(1)}(\rho)\right]\hat{e}_\phi\,,$$

$$\boldsymbol{N}_{o1n} = \frac{h_n^{(1)}(\rho)}{\rho}\sin(\phi)n(n+1)\sin(\theta)\,\pi_n(\theta)\hat{e}_r + \sin(\phi)\,\tau_n(\theta)\frac{1}{\rho}\frac{\partial}{\partial\rho}\left[\rho h_n^{(1)}(\rho)\right]\hat{e}_\theta \qquad (34d)$$

$$+ cos(\phi)\,\pi_n(\theta)\frac{1}{\rho}\frac{\partial}{\partial\rho}\left[\rho h_n^{(1)}(\rho)\right]\hat{e}_\phi\,.$$

Here, $h_n^{(1)}$ is the Hankel function of first kind. The angle-dependent functions are defined as:

$$\pi_n(\theta) = \frac{P_n^1(cos\,\theta)}{sin\,\theta}\,, \qquad \tau_n(\theta) = \frac{\partial P_n^1(cos\,\theta)}{\partial\theta}\,, \qquad (35)$$

where P_n^m are the Legendre polynomials with $m \in \{0,1,\dots\}$ and $n \in \{m, m+1,\dots\}$.

To mimic the scanning of the gold tip across the $MAPbI_3$ nanoparticle surface, we consider a spherical particle of radius a that is centered at the origin. We calculate the field distributions in the xy-plane at a) a height of $z = 1$ nm at positions outside the particle and b) at a height of $z = \sqrt{a^2 - x^2 - y^2} + 1$ nm at the surface of the particle. The field enhancement of the scattered field $|\boldsymbol{E}_S|/E_0$ is plotted in Fig. S4 for all three Cartesian vector components.

To rationalize the origin of the Fano resonance seen in Fig. 3 of the main manuscript, we analyze the wavelength dependence of the near-field distributions of the dipolar and quadrupolar modes of the particle in Fig. S5. Figure S5a-d shows cross sections for the dominant z-component of the optical near field along a line at $y = 0$ in the xy −plane. The near-field amplitudes are taken again at a distance of 1 nm from the surface of the nanoparticle, as in Fig. S4 and thus follow the height profile of the nanoparticle. The wavelength dependence of the resulting amplitude $|\boldsymbol{E}_{s,z}|$ and spectral phases $\phi_{s,z}$ are shown depicted for the ED in Fig. S5a,b while those calculated for the MQ

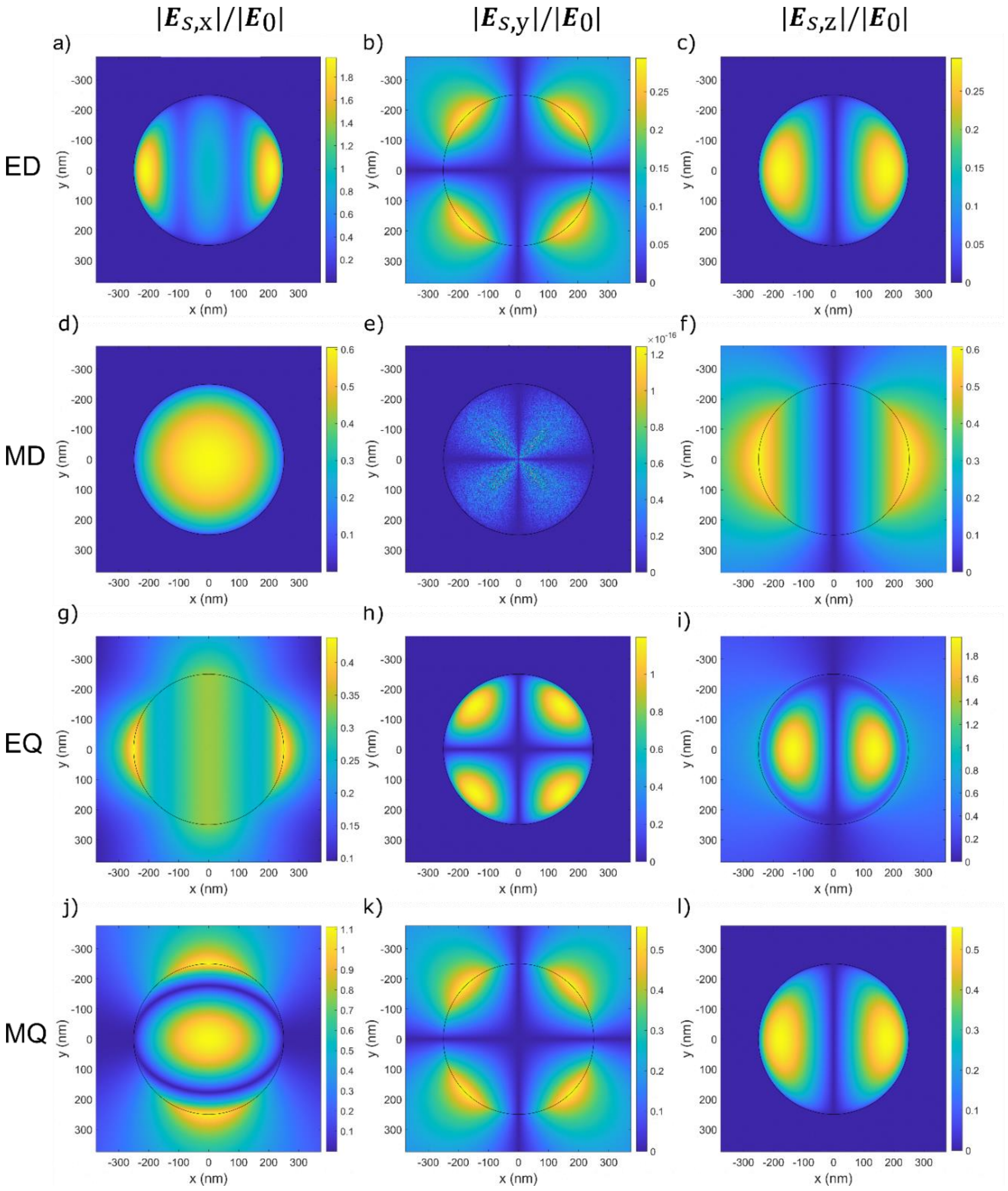

Figure S5. Normalized vectorial electric field components of the scattered near field $|\boldsymbol{E}_S|/|E_0|$ at the surface of a $MAPbI_3$ sphere with radius of 250 nm as obtained from Mie scattering theory. The field distributions are calculated at a wavelength of 820 nm for a refractive index of $MAPbI_3$ of n_1 = 2.43+0.024i at 820 nm wavelength. The field distributions are shown for the electric dipole mode (ED) in a)-c), for the magnetic dipole mode (MD) in d)-f), for the electric quadrupole mode (EQ) in g)-i) and for the magnetic quadrupole mode (MQ) in j)-l), respectively.

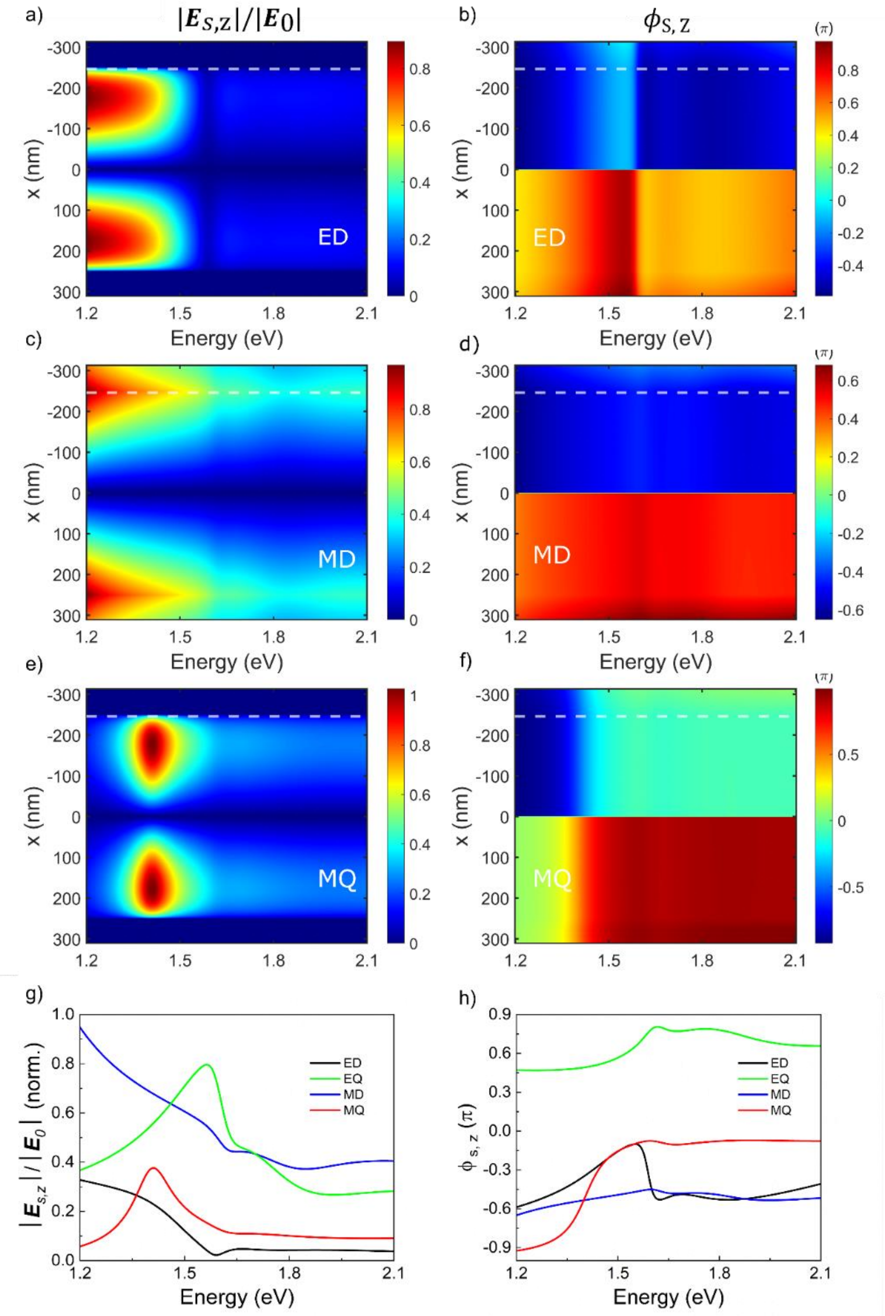

Figure S6. Cross sections of the z-component of scattered electric field at the surface of a $MAPbI_3$ sphere with radius of 250 nm as obtained from Mie theory. The cross sections are taken along a line at y=0 and follow the height profile of the particle. a-f) Amplitude and spectral phase of the electric dipole (ED), magnetic dipole (MD) and magnetic quadrupole (MQ) modes as a function of excitation wavelength. g-h) Amplitude and spectra phase of the z-component of the scattered electric field close to the edge, taken at the position marked as a dashed line in a)-f). A phase difference of π between the MD and EQ mode causes destructive interference between the modes.

mode are shown in Fig. S5c,d. The wavelength dependence of the amplitude and phase of all four relevant Mie modes in Fig. S5e,f at the position at the rim of the particle that is marked by a dashed line in Fig. S5a-d. A crosscut at $x = -245.8\ nm$ showing the amplitude and phase of all 4 relevant modes is depicted in Fig. S6e,f, respectively. A phase difference of π between the MD and EQ mode causes destructive interference between the modes in near-field measurements.

8. FDTD simulation of $MAPbI_3$ particles

The finite-difference time-domain (FDTD) method is used to carry out simulations of optical near fields of half-spherical $MAPbI_3$ particles placed on a substrate. All calculations are carried using the 3D electromagnetic simulator FDTD in Lumerical (Lumerical Inc.) A half-sphere composed $MAPbI_3$ is modeled by using the refractive index shown in Figure S1 and is placed on a substrate with constant refractive index of 1.5 (glass). This structure is illuminated by a linearly polarized plane wave in a total-field scattered-field (TFSF) configuration. Transmission monitors surrounding the structure are used to calculate both the scattering- and absorption cross-section. A three-dimensional field monitor is used to record the spectrally dependent field distribution in the vicinity of the particle. The three-dimensional field distribution is mapped onto a two-dimensional plane representing the surface that is scanned in the experiment. The setup of the FDTD simulations is shown in Figure S6 with a total size of the simulation box of 3x3x3 μm^3. Adaptive meshing is applied with a mesh size of 12 nm in free space down to 2 nm around the $MAPbI_3$ particle. Symmetric and anti-symmetric boundary conditions are used to reduce the computational effort, together with PML boundary conditions on the remaining sides of the simulation box.

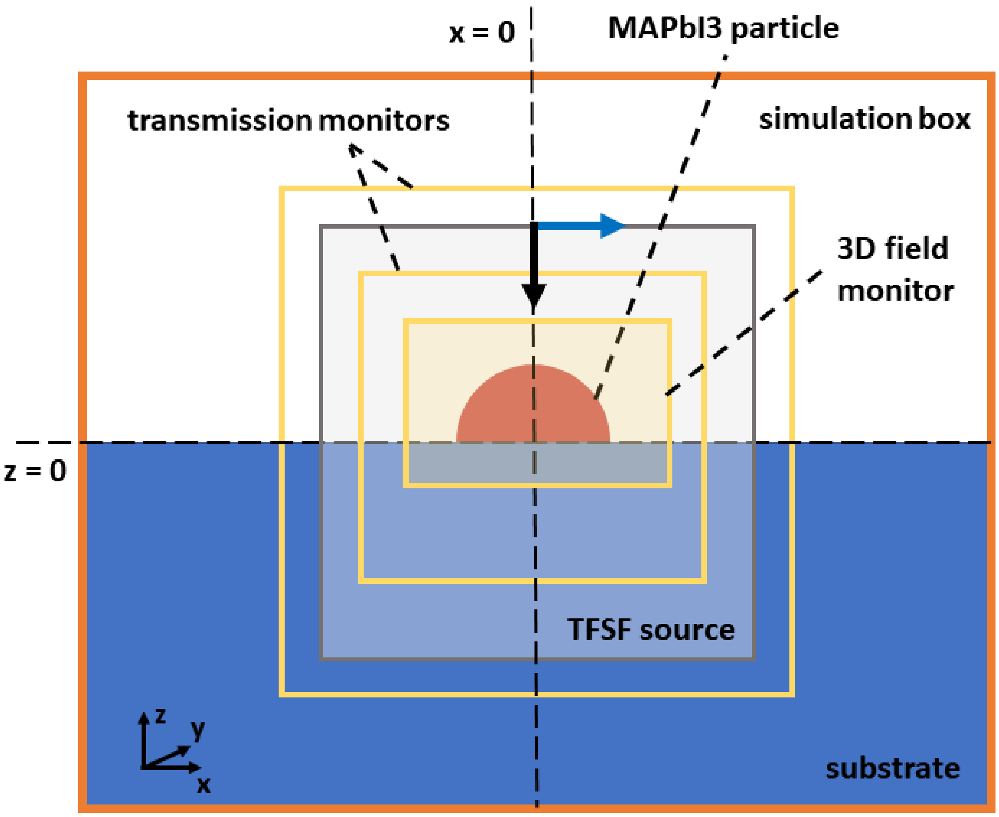

Figure S7. Setup of the FDTD simulations. The $MAPbI_3$ particle (red) is modelled as a half-sphere and placed on a substrate (blue, lower half-space). The particle is illuminated by a linearly polarized plane wave in a total-field scattered-field (TFSF) configuration (gray shaded box). Transmission monitors (yellow) are used to measure the scattered and absorbed fields. A 3D field monitor records the spectrally dependent field in the vicinity of the $MAPbI_3$ particle. Symmetric and anti-symmetric boundary conditions are applied together with PML boundary conditions. The simulation box (orange) has a size of 3x3x3 μm^3.

References

(1) Dmitriev, P. A.; Makarov, S. V.; Milichko, V. A.; Mukhin, I. S.; Gudovskikh, A. S.; Sitnikova, A. A.; Samusev, A. K.; Krasnok, A. E.; Belov, P. A. Laser fabrication of crystalline silicon nanoresonators from an amorphous film for low-loss all-dielectric nanophotonics. *Nanoscale* **2016**, *8* (9), 5043-5048, 10.1039/C5NR06742A.
(2) Tiguntseva, E. Y.; Baranov, D. G.; Pushkarev, A. P.; Munkhbat, B.; Komissarenko, F.; Franckevičius, M.; Zakhidov, A. A.; Shegai, T.; Kivshar, Y. S.; Makarov, S. V. Tunable Hybrid Fano Resonances in Halide Perovskite Nanoparticles. *Nano Lett.* **2018**, *18* (9), 5522-5529.
(3) Jeon, N. J.; Noh, J. H.; Kim, Y. C.; Yang, W. S.; Ryu, S.; Seok, S. I. Solvent engineering for high-performance inorganic–organic hybrid perovskite solar cells. *Nat. Mater.* **2014**, *13* (9), 897-903.

(4) Phillips, L. J.; Rashed, A. M.; Treharne, R. E.; Kay, J.; Yates, P.; Mitrovic, I. Z.; Weerakkody, A.; Hall, S.; Durose, K. Dispersion relation data for methylammonium lead triiodide perovskite deposited on a (100) silicon wafer using a two-step vapour-phase reaction process. *Data in Brief* **2015**, *5*, 926-928.
(5) Esmann, M.; Becker, S. F.; Witt, J.; Zhan, J.; Chimeh, A.; Korte, A.; Zhong, J.; Vogelgesang, R.; Wittstock, G.; Lienau, C. Vectorial near-field coupling. *Nat. Nanotechnol.* **2019**, *14* (7), 698-704.
(6) Esmann, M.; Chimeh, A.; Korte, A.; Zhong, J.-H.; Stephan, S.; Witt, J.; Wittstock, G.; Talebi, N.; Lienau, C. Plasmonic nanofocusing spectral interferometry. *Nanophotonics* **2020**, *9* (2), 491-508.
(7) Zhong, J. H.; Chimeh, A.; Korte, A.; Schwarz, F.; Yi, J. M.; Wang, D.; Zhan, J. X.; Schaaf, P.; Runge, E.; Lienau, C. Strong Spatial and Spectral Localization of Surface Plasmons in Individual Randomly Disordered Gold Nanosponges. *Nano Lett.* **2018**, *18* (8), 4957-4964.
(8) Schmidt, S.; Piglosiewicz, B.; Sadiq, D.; Shirdel, J.; Lee, J. S.; Vasa, P.; Park, N.; Kim, D.-S.; Lienau, C. Adiabatic Nanofocusing on Ultrasmooth Single-Crystalline Gold Tapers Creates a 10-nm-Sized Light Source with Few-Cycle Time Resolution. *Acs Nano* **2012**, *6* (7), 6040-6048.
(9) Lalor, É.; Wolf, E. Exact Solution of the Equations of Molecular Optics for Refraction and Reflection of an Electromagnetic Wave on a Semi-Infinite Dielectric*. *J. Opt. Soc. Am.* **1972**, *62* (10), 1165-1174.
(10) Knoll, B.; Keilmann, F. Enhanced dielectric contrast in scattering-type scanning near-field optical microscopy. *Opt. Commun.* **2000**, *182* (4-6), 321-328.
(11) Hillenbrand, R.; Keilmann, F. Complex optical constants on a subwavelength scale. *Phys. Rev. Lett.* **2000**, *85* (14), 3029-3032.
(12) Raschke, M. B.; Lienau, C. Apertureless near-field optical microscopy: Tip-sample coupling in elastic light scattering. *Appl. Phys. Lett.* **2003**, *83* (24), 5089-5091.
(13) Hecht, B.; Novotny, L. Far-field Green functions. In *Principles of Nano-Optics*, 2 ed.; Cambridge University Press, 2012; pp 543-544.
(14) Reynaud, F.; Salin, F.; Barthelemy, A. Measurement of phase shifts introduced by nonlinear optical phenomena on subpicosecond pulses. *Opt. Lett.* **1989**, *14*, 3.
(15) Lepetit, L.; Cheriaux, G.; Joffre, M. Linear techniques of phase measurement by femtosecond spectral interferometry for applications in spectroscopy. *J. Opt. Soc. Am. B* **1995**, *12* (12), 2467-2474.
(16) Ocelic, N.; Huber, A.; Hillenbrand, R. Pseudoheterodyne detection for background-free near-field spectroscopy. *Appl. Phys. Lett.* **2006**, *89* (10), 101124.
(17) Keilmann, F.; Hillenbrand, R. Near-field microscopy by elastic light scattering from a tip. *Philos. Trans. R. Soc., A* **2004**, *362* (1817), 787-805.
(18) Bohren, C. F.; Huffman, D. R. *Absorption and Scattering of Light by Small Particles*; Wiley-VCH, 1998. DOI: 10.1002/9783527618156.
(19) Schäfer, J.; Lee, S. C.; Kienle, A. Calculation of the near fields for the scattering of electromagnetic waves by multiple infinite cylinders at perpendicular incidence. *J. Quant. Spectrosc. Radiat. Transfer* **2012**, *113* (16), 2113-2123.
